# Phenomic landscape and pharmacogenomic implications for HLA region in a Taiwan Han Chinese population

**DOI:** 10.1186/s40364-024-00591-z

**Published:** 2024-05-03

**Authors:** Wan-Hsuan Chou, Lu-Chun Chen, Henry Sung-Ching Wong, Ching-Hsuan Chao, Hou-Wei Chu, Wei-Chiao Chang

**Affiliations:** 1 Department of Clinical Pharmacy, School of Pharmacy, Taipei Medical University, Taipei, Taiwan; 2 Institute of Biomedical Sciences, Academia Sinica, Taipei, Taiwan; 3 Master Program in Clinical Genomics and Proteomics, School of Pharmacy, Taipei Medical University, Taipei, Taiwan; 4 Integrative Research Center for Critical Care, Wan Fang Hospital, Taipei Medical University, Taipei, Taiwan; 5 Department of Pharmacy, Wan Fang Hospital, Taipei Medical University, Taipei, Taiwan; 6 Department of Pharmacology, National Defense Medical Center, Taipei, Taiwan

**Keywords:** Human Leukocyte Antigen, HLA, Major histocompatibility complex, MHC, Phenome-wide association study

## Abstract

**Background:**

The human leukocyte antigen (HLA) genes, exhibiting significant genetic diversity, are associated with susceptibility to various clinical diseases and diverse in drug responses. High costs of HLA sequencing and the population-specific architecture of this genetic region necessitate the establishment of a population-specific HLA imputation reference panel. Moreover, there is a lack of understanding about the genetic and phenotypic landscape of HLA variations within the Taiwanese population.

**Methods:**

We created models for a Taiwanese-specific HLA imputation reference panel. These models were trained with the array genotype data and HLA sequencing data from 845 Taiwanese subjects. HLA imputation was applied for 59,448 Taiwanese subjects to characterize the HLA allele and haplotype frequencies. Additionally, a phenome-wide association study (PheWAS) was conducted to identify the phenotypes associated with HLA variations. The association of the biallelic HLA variants with the binary and quantitative traits were evaluated with additive logistic and linear regression models, respectively. Furthermore, an omnibus test with likelihood-ratio test was applied for each HLA amino acid position in the multiallelic HLA amino acid polymorphisms to compare the difference between a fitted model and a null model following a χ2 distribution of n-1 degree of freedom at a position with n residues. Finally, we estimated the prevalence of adverse drug reactions (ADR)-related HLA alleles in the Taiwanese population.

**Results:**

In this study, the reference panel models displayed remarkable accuracy, with averages of 99.3%, 98.9%, and 99.1% for 2-, 4-, 6-digit alleles of the eight classical HLA genes, respectively. For PheWAS, a total of 18,136 significant associations with HLA variants across 26 phenotypes are identified (*p* < 5×10^-8^), highlighting the pleiotropy feature of the HLA region. Among the independent signals, 15 are novel, including the association of *HLA-B* pos 138 variation with ankylosing spondylitis (AS), and rs9266290 and rs9266292 with allergy. Through an analysis spanning the entire HLA region, we identified clusters of phenotype correlations. Finally, the carriers of pharmacogenomic related HLA alleles, including HLA-C*01:02 (35.86%), HLA-B*58:01 (20.9%), and HLA-B*15:02 (8.38%), were characterized in the Taiwanese general population.

**Conclusions:**

We successfully delivered the HLA imputation for 59,448 Taiwanese subjects and characterized the genetic and phenotypic landscapes of the HLA variations. In addition, we quantified the estimated prevalence of the ADR-related HLA alleles in the Taiwanese population. The developed HLA imputation reference panel could be used for estimation of population HLA allele frequencies, which can facilitate further studies in the role of HLA variants in a wider range of phenotypes in the population.

**Supplementary Information:**

The online version contains supplementary material available at 10.1186/s40364-024-00591-z.

## Background

The human leukocyte antigen (HLA) corresponds to a cluster of genes located on the chromosome 6p21 that encode the major histocompatibility complex (MHC) in humans [1]. These genes play a pivotal role in differentiating self and non-self by presenting a vast amount of antigen peptides to T cells [2]. The HLA genes exhibit an incredible diversity, with more than 35,000 alleles [3]. This intensive polymorphism enables the immune system to fight against a variety of pathogens and diseases. Indeed, the HLA region has been considered as a critical area associated with a wide spectrum of diseases, including type I diabetes, rheumatoid arthritis (RA), and ankylosing spondylitis (AS) [4, 5]. Moreover, the HLA variations have been repeatedly alerted to adverse drug reactions, including abacavir hypersensitivity and carbamazepine-induced Stevens–Johnson syndrome [6–9]. These studies indicate the pleiotropy of HLA and highlight the importance to investigate the HLA variations in different phenotypes.

Phenome-wide association studies (PheWAS) is research tool to investigate the association of genetic variances across extensive phenotypes. PheWAS is a transposition of GWAS. Unlike GWAS that test the associations of the genetic variants across the genome with a specific phenotype, PheWAS aims to identify the phenotypes associated with certain genetic variants [10, 11]. GWAS has been widely applied to identify disease predisposition genes and pathways involved in complex human traits [10]. On the other hand, PheWAS can be applied to study the shared mechanisms across different traits and to identify the target diseases for drug repurposing [11]. In addition, PheWAS has become feasible and increasingly popular with the emergence of biobanks of densely phenotyped cohorts or link to the participants’ electronic health records. Taiwan biobank TWB has enrolled more than 150 thousand individuals since its establishment in 2012. The comprehensive phenotypes collected with questionnaires and physical and biochemical examinations make it an ideal resource for phenome-wide investigation [12].

Genetic and phenotypic characteristics within the HLA region have been investigated in European, Japanese and Korean populations [13–16]. However, the structure of linkage disequilibrium (LD) is extremely complex in the MHC region and the complexity leads to diverse haplotypes across different populations [4]. In addition, previous genomic study of TWB revealed a distinct cluster with unique HLA pattern, indicating high degree of LD between HLA haplotype A*33:03-B*58:01 [17]. Furthermore, previous studies showed that certain HLA-phenotype associations can be replicated, while others are population specific [13–16]. These give prominence to the need of population specific investigation in HLA region.

Direct sequencing-based typing (SBT) is the gold standard for HLA typing in clinical settings. Next-generation sequencing (NGS) -based typing has shown high concordance with SBT [18]. However, sequencing-based HLA typing is labor-intensive, expensive, and not widely applied in large cohorts. On the other hand, extensive genotype data in huge cohorts has been collected with the establishment of biobanks around the world. Furthermore, a variety of strategies to impute HLA alleles from genotype data have been developed to enable the investigation of HLA variations in large cohorts [19]. To extend the use of HLA imputation and to account for the population diversity in HLA region, it is essential to develop population-specific HLA imputation reference panels in different populations.

Recently, Y.H. Huang et al. constructed a Taiwanese-specific HLA imputation reference. This reference panel was applied to the genotype data from 23,972 Taiwanese subjects. Importantly, the results successfully validated the association of HLA variations with RA in the Taiwanese population [20]. However, the investigation of comprehensive phenotypic landscape of the HLA variants was not conducted in Taiwan Han Chinese population yet. In addition, the reference panel constructed in that study was based upon the genotype data of TWBv1 array, which was the first custom single-nucleotide polymorphism (SNP) array designed for genotyping in Taiwan Biobank (TWB) [12, 20]. The TWB has designed another SNP array, namely the TWBv2 array, to include rare coding risk alleles and more variants across the HLA region, and has released an expanded cohort of TWBv2 genotype data [12, 21]. Given these factors, we aimed to develop a Taiwanese-specific HLA imputation reference panel based upon the TWBv2 array for HLA imputation in an expanded cohort of Taiwan Han Chinese population. Moreover, with the imputed HLA alleles, we aimed to investigate the genetic features of the HLA region and the phenotypic characteristics associated with HLA variations in a Taiwan Han Chinese population.

## Methods

### Study design

**Fig. 1:**
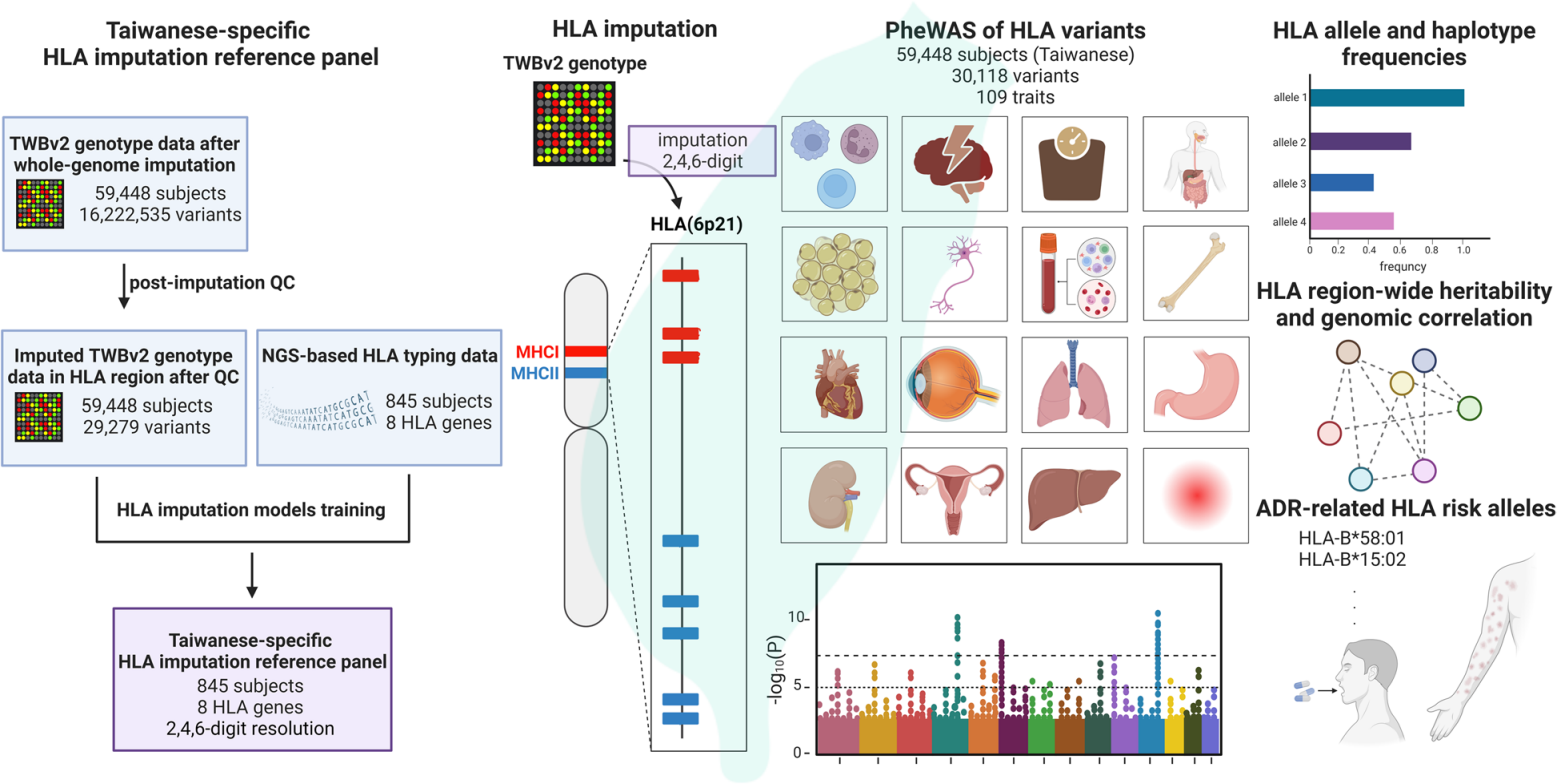
Schematic overview of this study

A schematic overview of the study is shown in Fig. 1 (created with BioRender.com). The Taiwan Biobank (TWB) data was harnessed in this study and the predominant majority of the TWB subjects are Han Chinese descents [12, 21]. To construct the Taiwanese-specific HLA imputation references using the TWB subjects with both the TWBv2 array genotype data and the next generation sequencing (NGS)-based HLA typing data. Furthermore, the reference panels were applied for HLA imputation on the TWB subjects with TWBv2 array genotype data. With this imputed cohort, we characterize the frequencies of HLA alleles and haplotypes in Taiwanese population. Moreover, a PheWAS was conducted to investigate the associations between HLA variations and 109 traits in 59,448 subjects. We also estimated the HLA region-wide heritability and genetic correlations of the phenotypes. Finally, we assessed the prevalence of adverse drug reactions (ADR)-related HLA alleles in the Taiwanese population. This study was conducted in accordance with The Code of Ethics of the World Medical Association. Deidentified data of human subjects was applied from the Taiwan Biobank, Academia Sinica. This study was approved by the Institutional Review Board of Taipei Medical University (TMU-JIRB N201906005). The access to and use of the Taiwan Biobank resource in this research was approved by the Ethics and Governance Council (EGC) of Taiwan Biobank (approval number: TWBR10906-03).

### Data resources and processing

In this study, individual genotype, sequencing-based HLA typing, and phenotype data of subjects enrolled to the TWB between 2012 and 2019 was used for analysis. Subjects without TWBv2 array genotype data were not included. Quality control, phasing and genome-wide imputation of the genotype data were conducted by TWB, as described in the previous study [12]. Overall, 16,222,535 variants in 68,948 subjects with the imputation INFO score > 0.3 were initially included in this study. Furthermore, post-genome-wide imputation quality control (QC) filtering was applied using PLINK 1.9. First of all, low quality variants meeting any of the following criteria were excluded: (1) variant call rate < 0.98; (2) minor allele frequency (MAF) < 0.05; (3) deviation from Hardy-Weinberg equilibrium (HWE) *p*-value < 1×10^-6^. Furthermore, subjects meeting any of the criteria below were excluded: (1) incorrect sex status; (2) sample call rate < 0.98; (3) outlying heterozygosity rate (outside 3 times of the standard deviation from the mean); (4) Identity by descent (IBD) > 0.1875. After these quality control, a total of 3,982,815 variants and 59,448 subjects remained. Finally, variants located on the MHC region, which is on chromosome 6, ranging from 28,510,120 to 33,480,577 bp based on GRCh38 (accessed in May 2021), were extracted for further analysis. Overall, 59,448 subjects and 29,279 variants, including 27,055 biallelic single nucleotide variations (SNVs) and 2224 indels, were included for analysis.

Amongst these subjects, 845 had NGS-based HLA typing data of eight classical HLA genes (*HLA-A*, *HLA-B*, *HLA-C*, *HLA-DRB1*, *HLA-DPA1*, *HLA-DPB1*, *HLA-DQA1*, and *HLA-DQB1*) available from TWB. The sequencing library was constructed with the NXType^TM^ NGS HLA typing kit (One Lambda, Inc., Canoga Park, CA) and the Ion Chef^TM^ system (Thermo Fisher Scientific, Waltham, MA). Furthermore, sequencing data was performed with the Ion S5^TM^ XL sequencer (Thermo Fisher Scientific, Waltham, MA). Finally, the HLA alleles corresponding to the IMGT/HLA database (version 3.29.0.1) were called using the TypeStream^TM^ software v.1.1.0 (One Lambda, Inc., Canoga Park, CA)[20].

Phenotypes were collected by TWB through physical examinations and a structured questionnaire for health-related information. Furthermore, the blood and urine specimens provided by the TWB participants were subjected to laboratory tests. Detailed procedure of phenotype collection was described in a previous study [12]. In this study, self-reported diseases based on the questionnaire were extracted as binary traits. Individuals with each self-reported disease were defined as cases, and the other subjects were the corresponding controls. For gynecological diseases, female controls were used. Furthermore, quantitative traits were extracted from the results of the physical and laboratory examinations. A total of 109 traits, including 55 binary traits (Supplementary table 1) and 54 quantitative (Supplementary table 2), were included in the association analysis.

### Construction of Taiwanese-specific HLA imputation reference panel

In this study, all 845 TWB subjects with both TWBv2 genotype data and NGS-based HLA typing data were included to construct the Taiwanese-specific HLA imputation reference panel. Taiwanese-specific HLA parameter estimates of two-, four-, and six-digits resolution for the eight classical HLA genes were generated from the 845 TWB subjects with both the TWBv2 array genotype and the HLA typing data using the HIBAG R package (version 1.26.1) [22, 23]. SNVs within the 500 kb flanking region of each HLA gene were included and 100 classifiers were adopted for the generation of each parameter estimate. Performance of each HLA parameter estimate was assessed via internal validation by randomly dividing the study subjects into a training set and a validation set at a 7:3 proportion. The parameter estimates generated from the training set were applied for HLA imputation in the validation set. The imputation accuracies were calculated by comparing the direct HLA typing results and the imputed HLA alleles with call threshold ≥ 0.5 for posterior probability in the validation set.

### HLA imputation and haplotype frequencies calculation

After construction of the Taiwanese-specific HLA reference panel, it was applied for HLA imputation on the 59,448 TWB subjects with TWBv2 array genotype data using the HIBAG R package (version 1.26.1) [22, 23]. External validation was carried out by comparing the distribution of HLA allele frequencies in our imputed cohort with those in other Taiwanese cohorts obtained from the allele frequency net database (AFND) [24]. The datasets included for comparisons were: (1) MJ Lai et al., consisted of 46682 subjects from the Tzu Chi Taiwan Marrow Donor Registry with *HLA-A*, *B*, and *DRB1* genotypes determined by the PCR sequence specific oligonucleotide probe (PCR-SSO), PCR sequence specific primer (PCR-SSP), and the sequencing methods [25]; (2) SH Wen et al., consisted of 710 subjects from the Tzu Chi Taiwan Cord Blood Bank with *HLA-A*, *B*, and *DRB1* genotypes determined by the PCR-SSP method [26]; and (3) PL Chen et al., consisted of 504 subjects with *HLA-A*, *B*, *C*, *DRB1*, and *DQB1* genotypes determined by the PCR-SSO and PCR-SSP methods [27]. Furthermore, haplotypes of the eight classical HLA genes were phased and the frequencies were calculated with haplo.stats (version 1.8.6) R package. In addition, haplotypes of five HLA genes (*HLA-A*, *HLA-C*, *HLA-B*, *HLA-DRB1*, and *HLA-DQB1*) were phased to compare the haplotype frequencies with the frequencies in a Han Chinese population [28].

### Phenome-Wide Association Study (PheWAS)

In addition to the biallelic SNVs and indels in the HLA region, the following types of HLA variants were included in the PheWAS analysis: (1) imputed HLA alleles in 2-, 4-, and 6-digit resolution; (2) HLA amino acid polymorphisms (e.g. *HLA-B* position 138); (3) biallelic HLA amino acid polymorphisms of the respective residue (e.g. *HLA-B* Q94). The amino acid sequences were translated from the imputed HLA alleles using the HIBAG R package (version 1.26.1) and the amino acid polymorphisms were identified from the amino acid sequences [22, 23]. Additional QC after the HLA imputation and amino acid translation were applied as follows: (1) for the HLA alleles and the biallelic HLA amino acid polymorphisms, variants with missing rate >0.05 or MAF < 0.01 were excluded; (2) for multiallelic HLA amino acid polymorphisms, variants with missing rate >0.05 were excluded. In this study, a total of 30,188 variants, including 27,055 biallelic SNVs, 2,224 indels, 269 HLA alleles, 437 biallelic HLA amino acid polymorphisms, and 203 multiallelic HLA amino acid polymorphisms, were included in the association analysis.

The association between the HLA variants and the phenotypes were evaluated using regressions under additive genetic models using REGENIE (version 2.2.4) [29]. The sex, age, age^2^, and the top 10 principal components (PCs) obtained from the genome-wide genotype data were included as covariates. The association between the biallelic HLA variants and the binary traits were evaluated with an additive logistic regression model. The quantitative traits were first regressed with the covariates. Furthermore, the association of the biallelic HLA variants with the inverse- normal transformed residuals were evaluated using an additive linear regression model. For the multiallelic HLA amino acid polymorphisms, an omnibus test with likelihood-ratio test was applied for each HLA amino acid position to compare the difference between a fitted model and a null model following a χ^2^ distribution of n-1 degree of freedom at a position with n residues. The genome-wide significant (GWS) threshold of *p* < 5×10^-8^ was used in this study. The biallelic SNVs and indels were annotated using ANNOVAR [30]. Furthermore, these variants were assigned to the eight classical HLA genes if they were in high LD of r^2^ ≥ 0.7 with any HLA allele or amino acid polymorphism on the corresponding HLA genes.

The forward-type conditional regression analysis was applied for the phenotypes with at least one GWS signal to identify the independent association signals using REGENIE (version 2.2.4) [29]. For each phenotype, the top signal and the corresponding HLA alleles and HLA amino acid polymorphisms in high LD (r^2^ ≥ 0.7) with it were included into the regression model as covariates. The new top signal identified and its high-LD variants were included as covariates in the next iterative step of the conditional regression. The iterative step was repeated until there was no additional independent signal detected. This modified forward type step-wise conditional analysis additionally include HLA polymorphisms, namely the HLA alleles and the HLA amino acid polymorphisms as covariates and could rule out the effect of the same HLA gene and condition the associations attributable to the HLA gene robustly [15, 16]. GWAS Catalog (Accession date: December 21, 2021) was queried to determine if the associations with the biallelic SNVs and indels were novel. The associations were considered novel if no significant signal was identified within 500 kb of the variant with the same phenotype in the GWAS Catalog. Literature review was conducted to determine if the associations of the phenotypes with the HLA alleles or the amino acid polymorphisms were novel.

The region-wide heritability estimates of each phenotype and the pairwise genetic correlations between the phenotypes accounting for the HLA variants (SNVs and indels in the HLA regions, the HLA alleles and the HLA amino acid polymorphisms) were calculated with the univariate and the bivariate Haseman-Elston (HE) regression using GCTA (version 1.93.2beta), respectively [31]. The genetic correlation network was visualized with the igraph R package (version 1.2.7) [32].

### Pharmacogenomics of HLA variations

The burden of adverse drug reactions (ADR)-related HLA alleles in the Taiwanese population was assessed by summarizing the proportion of carriers of selected ADR-related HLA alleles in the imputed cohort. The selected ADR-related HLA alleles were collected through literature search [6, 7, 33, 34].

## Results

### Internal validation of the Taiwanese HLA imputation reference panel

**Fig. 2:**
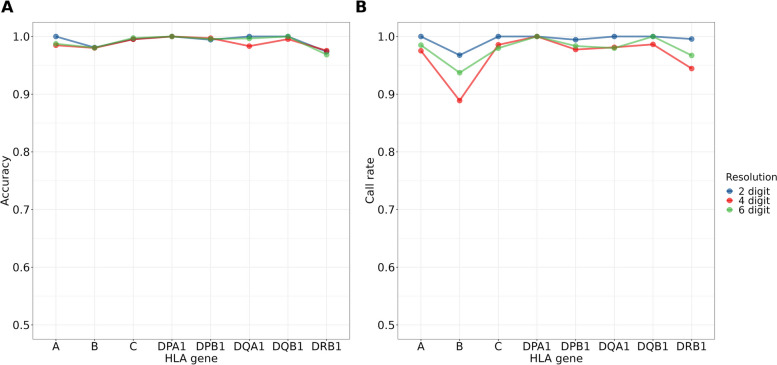
Internal validation imputation accuracy (**A**) and call rate (**B**) of the Taiwanese-specific HLA reference panels. The internal validation imputation accuracy and call rate were calculated after applying a call threshold ≥ 0.5 (*n*=845)

We constructed a Taiwanese-specific HLA imputation reference panel using array-based (TWBv2) genome-wide genotype data and NGS-based HLA typing data from 845 subjects in TWB. While seven subjects had missing data of self-reported parent originality, all the remaining 838 subjects in the reference panel are self-reported Han Chinese descendants with parents that are either Taiwanese Minnan, Taiwanese Hakka, or Chinese immigrants (Supplementary table 3). This panel was composed of 24 models to predict HLA types of the eight classical HLA genes in 2-, 4-, and 6- digit resolutions from genotype data. A total of 581 2-, 4-, 6-digit HLA alleles derived from the NGS-based HLA typing data were subjected to the model training. As shown in Fig. 2A, the reference panel exhibited high imputation accuracies for eight HLA genes (averages of 99.3%, 98.9%, and 99.1% for 2-, 4-, 6-digit HLA alleles, respectively). Call rates of the panel were also assessed for these HLA genes, with averages of 99.5%, 96.7%, and 97.9% in 2-, 4-, 6-digit models, respectively (Fig. 2B).

### HLA frequencies in the Taiwanese population

**Fig. 3:**
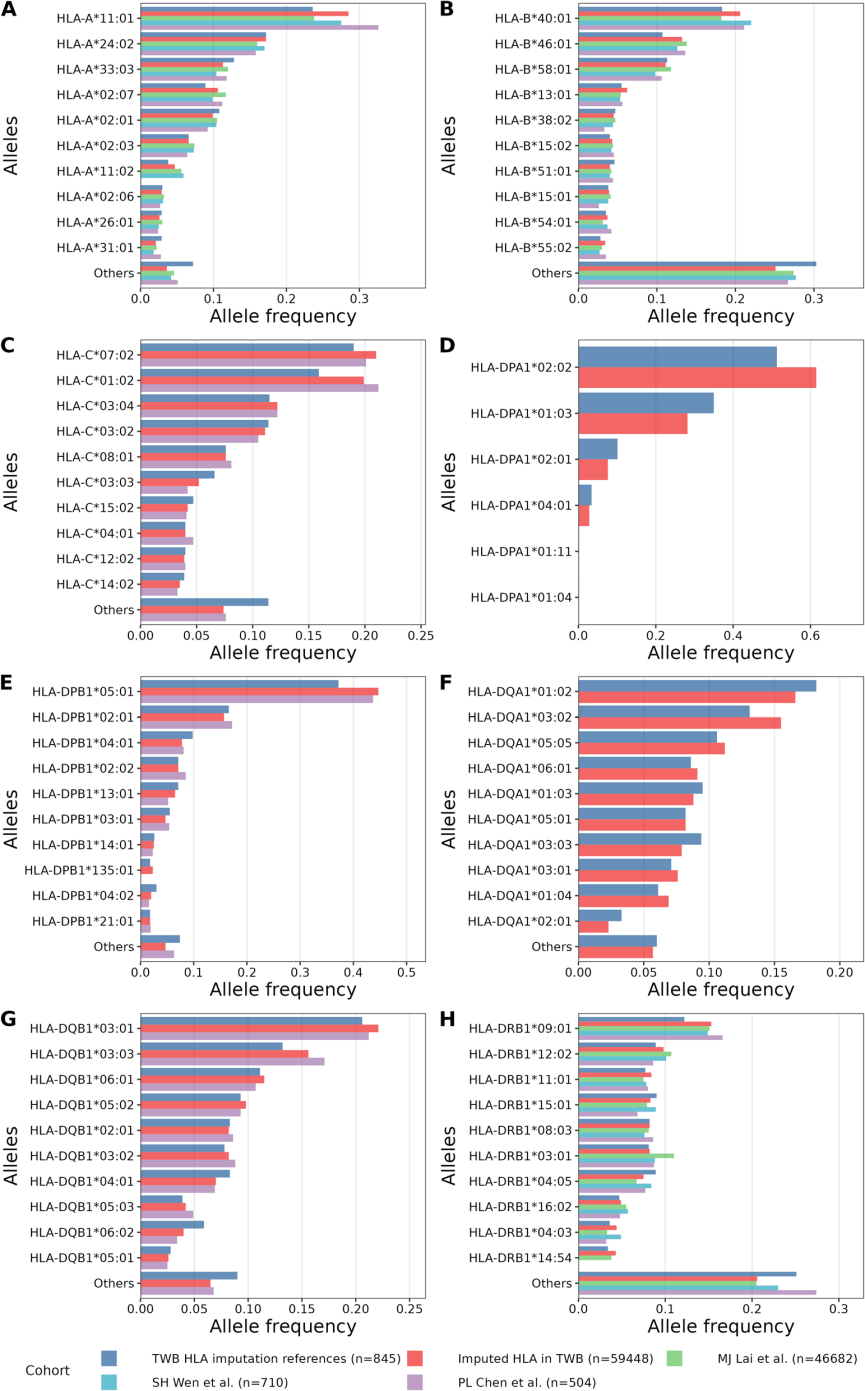
Comparisons of HLA allele frequencies in the TWB cohorts with the external Taiwanese datasets. Four-digit allele frequencies of *HLA-A* (**A**), *HLA-B* (**B**), *HLA-C* (**C**), *HLA-DPA1* (**D**), *HLA-DPB1* (**E**), *HLA-DQA1* (**F**), *HLA-DQB1* (**G**), *HLA-DRB1* (**H**) are shown in this figure. Top ten alleles of each gene in the TWB cohort after HLA imputation were extracted and other alleles were grouped into the “other” allele for visualization and comparison with the other datasets

The mean age of the TWB HLA imputed cohort (*n* = 59448) was 50.5 years old (50.51±10.58), and nearly one third of the subjects were male (31.7%). The distribution of HLA allele frequencies in TWB cohorts by NGS-based HLA typing data (*n*=845) and by HLA imputation of genome-wide genotyping data (*n*=59,448) were similar across different resolutions (Supplementary figure 1, Supplementary table 4). Furthermore, we compared the HLA allele distributions of the TWB cohorts with additional three Taiwanese cohorts from AFND. The allele frequencies of *HLA-A*, *HLA-B*, *HLA-C*, *HLA-DPB1*, *HLA-DQB1*, and *HLA-DRB1* genes exhibited similar distributions in our TWB cohorts and in other Taiwanese cohorts (Fig. 3, Supplementary table 5, Supplementary dataset 1).

To further evaluate the performance of the HLA imputation and to characterize the HLA genetic architecture in the Taiwanese population, we analyzed HLA haplotypes and compared the frequencies of the TWB cohort with a Chinese population, which is genetically close to the Taiwanese population. As shown in the Supplementary table 6, the most common four-digit HLA haplotype of eight classical HLA genes was A*33:03-C*03:02-B*58:01-DRB1*03:01-DQA1*05:01-DQB1*02:01-DPA1*01:03-DPB1*04:01, with a frequency of 3.85% in the Taiwanese population. Further comparison of HLA haplotypes of five HLA genes shared by both populations showed that the frequencies of HLA haplotypes in our TWB cohort were similar with that in the Han Chinese population. However, the HLA haplotype frequencies were observed to be slightly divergent across different Han Chinese subpopulations. The distribution of the HLA haplotype frequencies in our TWB cohort were more similar to the southern Han Chinese subpopulation. In both Taiwanese population and southern Han Chinese subpopulation, A*33:03-C*03:02-B*58:01-DRB1*03:01-DQB1*02:01 and A*02:07-C*01:02-B*46:01-DRB1*09:01-DQB1*03:03 were identified as the most common HLA haplotypes (Supplementary table 7).

### Phenome-Wide Association Study

To investigate the phenotypic landscape within the HLA region for identifying phenotypes associated with HLA variants, we conducted a PheWAS using the TWB dataset with imputed HLA alleles. A total of 109 phenotypes were included in the analysis. Among them, 55 phenotypes as binary traits were classified into 12 categories, namely immune-related disease, metabolic disease, cardiovascular disease, kidney disease, digestive disease, mental disease, neurologic disease, ophthalmic disease, gynecological disease, bone disease, respiratory disease, and symptom (Supplementary table 1). Furthermore, another 54 phenotypes as quantitative traits fall into 8 categories, which were anthropometric, metabolic, cardiovascular, kidney-related, liver-related, hematological, bone-related, and respiratory (Supplementary table 2).

**Fig. 4:**
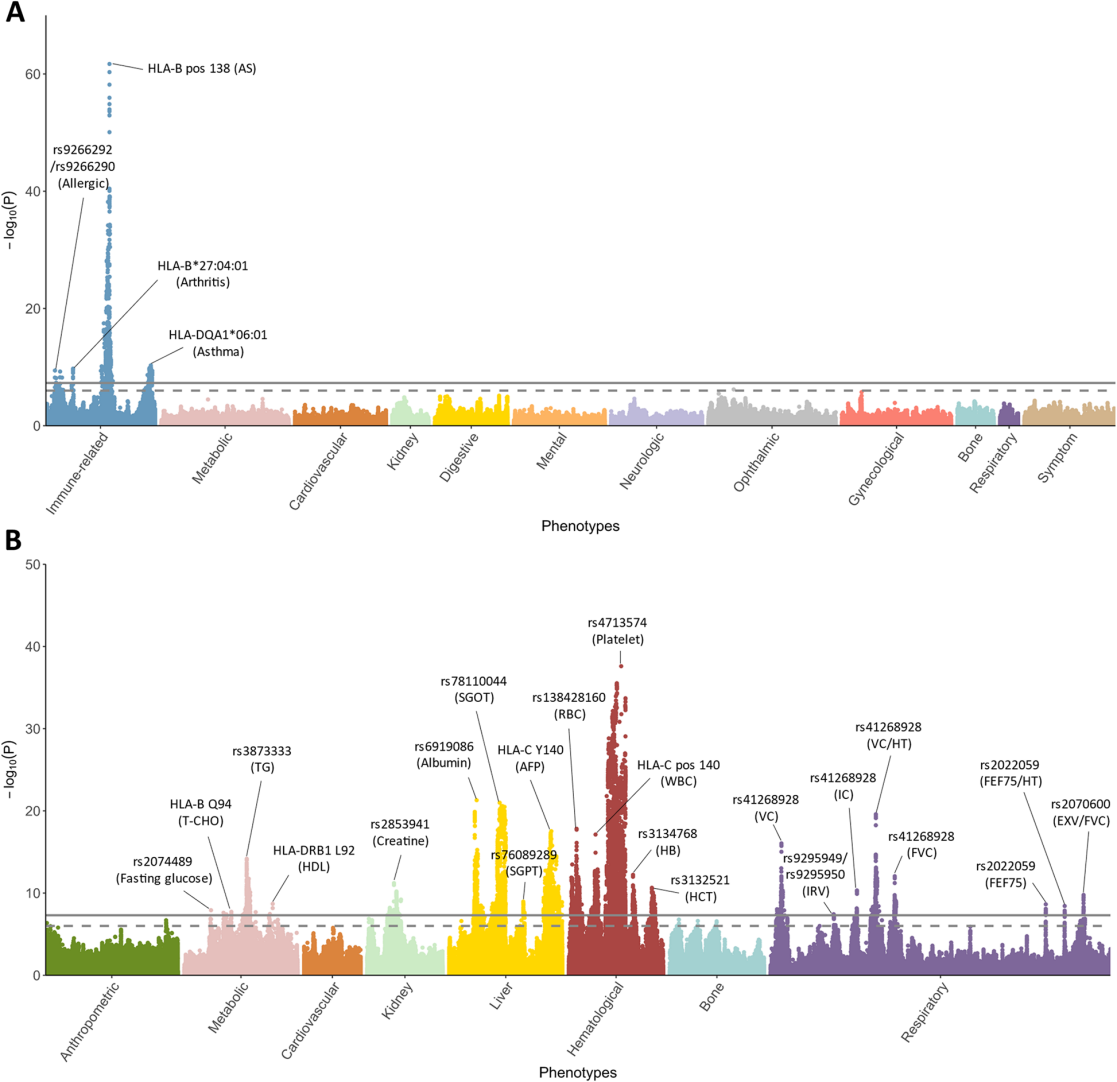
Associations of HLA variations with binary traits (**A**) and quantitative traits (**B**). The solid lines indicate the threshold of genome-wide significance (*p*-value < 5×10^-8^); the dashed lines indicate the threshold of suggestive significance (*p*-value < 10^-6^); AS, Ankylosing Spondylitis; T-CHO, Total cholesterol; TG, Triglyceride; HDL, High density lipoprotein cholesterol; SGOT, Serum glutamic oxaloacetic transaminase; SGPT, Serum glutamic pyruvic transaminase; AFP, Alpha-Fetoprotein; RBC, Red blood cell count; WBC, White blood cell count; HB, Hemoglobin; HCT, Hematocrit; VC, Vital capacity; IRV, Inspiratory Reserve Volume; IC, Inspiratory capacity; VC/HT, Vital capacity/Height ratio; FVC, Forced vital capacity; FEF75, 75% of Forced expiratory flow; FEF75/HT, 75% of Forced expiratory flow/height ratio; EXV/FVC, Extrapolated Volume/ Forced vital capacity ratio

In the PheWAS, a total of 18,136 GWS associations with HLA variants were identified (Supplementary table 1-2, Supplementary dataset 2). These associations were found across 26 phenotypes, approximately one quarter of the 109 phenotypes tested, demonstrating pleiotropic characteristics of the HLA region in the Taiwanese population. After conditional analysis, these phenotypes showed at least one independent signal (Table 1). As shown in Fig. 4A and Table 1, four of the 26 phenotypes were binary traits. All of them were categorized as immune-related diseases, including drug allergy, arthritis, asthma, and AS. The most significant signal was the association between the *HLA-B* variation on the amino acid 138 and AS (*p* = 1.94×10^-62^). By contrast, the other 22 HLA variant-associated phenotypes were quantitative traits and fall into diverse categories. Most of these phenotypes were in the respiratory examination category (*n*=8), and the others were based on hematological examination (*n*=5), liver function (*n*=4), metabolic examination (*n*=4), and kidney function (*n*=1) (Fig. 4B, Table 1). After the forward-type conditional regression analysis, additional five independent association signals of RBC (*n*=3), WBC (*n*=1), and platelet (*n*=1) were identified. As a result, our PheWAS identified a total of 18,136 significant association signals, including 31 independent signals and another two variants that were in complete LD with phenotype-associated top variants (Table 1). Among the independent and top signals, 18 associations between HLA variants and phenotypes were published elsewhere (Table 1, Supplementary table 8). For example, HLA-DQA1*06:01 is the top signal associated with asthma in the Taiwanese cohort, which is consistent with a previous study of Chinese asthma families [35]. The rest of 15 associations were novel and not reported by the GWAS Catalog or previous studies (Table 1, Supplementary table 8).

**Table 1:** Top and independent signals with genome-wide significance in the HLA region

**Category**	**Traits**	**Variants**	**Position (hg38)**	**Alleles (alt/ref)**	**HLA genes with high LD star allele or amino acid polymorphisms**	**Allele frequencies**^**d**^	**Effect size**^**e**^	**P value**^**f**^	**Novel**^**g**^
Immune-related disease	Allergic	rs9266290	31361306	A/G	*HLA-B*	0.275,0.248	1.150(1.101-1.201)	3.80×10^-10^	Yes
		rs9266292^a^	31361316	A/G	*HLA-B*	0.275,0.248	1.150(1.101-1.201)	3.80×10^-10^	Yes
	Arthritis	HLA-B*27:04:01	-	-	*HLA-B*	0.040,0.026	1.597(1.394-1.830)	1.83×10^-10^	Yes
	Asthma	HLA-DQA1*06:01	-	-	*HLA-DQA1*	0.064,0.092	0.675(0.596-0.763)	5.06×10^-11^	No
	AS	*HLA-B* pos 138^b^	-	-	*HLA-B*	-	-	1.94×10^-62^	Yes
Metabolic QTL	Fasting glucose	rs2074489	31272351	T/C	*-*	0.331	0.035(0.006)	1.21×10^-8^	Yes
	HDL	*HLA-DRB1*-L92	-	-	*HLA-DRB1*	0.342	0.038(0.006)	2.02×10^-9^	Yes
	T-CHO	*HLA-B*-Q94	-	-	*HLA-B*	0.226	-0.039(0.007)	1.89×10^-8^	Yes
	TG	rs3873333	30928275	C/T	*-*	0.431	0.046(0.006)	6.74×10^-15^	No
Kidney-related QTL	Creatinine	rs2853941	31281452	C/T	*HLA-C*	0.414	0.040(0.006)	6.15×10^-12^	No
Liver-related QTL	Albumin	rs6919086	31335150	A/G	*-*	0.449	-0.056(0.006)	5.01×10^-22^	No
	SGOT	rs78110044	32559333	C/G	*HLA-DRB1,HLA-DQA1, HLA-DQB1*	0.085	-0.099(0.010)	1.02×10^-21^	No
	SGPT	rs76089289	32997926	G/A	*HLA-DRB1,HLA-DQA1, HLA-DQB1,HLA-DPB1*	0.065	-0.072(0.012)	1.10×10^-9^	No
	AFP	*HLA-C*-Y140	-	-	*HLA-C*	0.374	-0.052(0.006)	2.94×10^-18^	Yes
Hematological QTL	RBC	rs138428160	31267408	A/AGTCAAGGTAACC	*-*	0.336	-0.054(0.006)	1.51×10^-18^	No
		*HLA-A*-V100^c^	-	-	*HLA-A*	0.789	-0.040(0.007)	2.39×10^-8^	Yes
	WBC	*HLA-C* pos 140^b^	-	-	*HLA-C*	-	-	7.50×10^-18^	Yes
		rs1056429^c^	31354106	A/G	*HLA-B*	0.331	-0.052(0.008)	9.57×10^-11^	No
	Platelet	rs4713574	32659261	G/C	*HLA-B, HLA-C, HLA-DRB1, HLA-DQA1, HLA-DQB1*	0.138	0.109(0.008)	2.49×10^-38^	No
		rs9394145^c^	33432001	T/C	*-*	0.309	0.061(0.006)	2.37×10^-21^	No
		rs3131002^c^	31124892	G/A	*-*	0.486	0.035(0.006)	6.93×10^-9^	No
		rs3094575^c^	29548025	T/C	*-*	0.421	0.035(0.006)	1.29×10^-8^	No
	HB	rs3134768	31239067	C/G	*HLA-C*	0.26	-0.048(0.007)	6.09×10^-13^	No
	HCT	rs3132521	31234903	T/C	*HLA-C*	0.261	-0.044(0.007)	2.25×10^-11^	No
Respiratory QTL	VC	rs41268928	32179380	C/G	*-*	0.225	-0.069(0.008)	8.99×10^-17^	Yes
	IC	rs41268928	32179380	C/G	*-*	0.224	-0.057(0.009)	4.75×10^-11^	Yes
	VC/HT	rs41268928	32179380	C/G	*-*	0.225	-0.076(0.008)	2.59×10^-20^	Yes
	FVC	rs41268928	32179380	C/G	*-*	0.225	-0.059(0.008)	8.83×10^-13^	No
	IRV	rs9295949	31078024	A/C	*-*	0.224	-0.048(0.009)	3.76×10^-8^	Yes
		rs9295950^a^	31078025	T/C	*-*	0.224	-0.048(0.009)	3.76×10^-8^	Yes
	EXV/FVC	rs2070600	32183666	T/C	*-*	0.221	0.053(0.008)	1.69×10^-10^	Yes
	FEF75	rs2022059	32188712	C/G	*-*	0.186	0.053(0.009)	2.30×10^-9^	Yes
	FEF75/HT	rs2022059	32188712	C/G	*-*	0.186	0.052(0.009)	3.72×10^-9^	Yes

*AS* Ankylosing Spondylitis, *T-CHO* Total cholesterol, *TG* Triglyceride, *HDL* High density lipoprotein cholesterol, *SGOT* Serum glutamic oxaloacetic transaminase, *SGPT* Serum glutamic pyruvic transaminase, *AFP* Alpha-Fetoprotein, *RBC* Red blood cell count, *WBC* White blood cell count, *HB* Hemoglobin, *HCT* Hematocrit, *VC* Vital capacity, *IRV* Inspiratory Reserve Volume, *IC* Inspiratory capacity, *VC/HT* Vital capacity/Height ratio, *FVC* Forced vital capacity, *FEF75* 75% of Forced expiratory flow, *FEF75/HT* 75% of Forced expiratory flow/height ratio, *EXV/FVC* Extrapolated Volume/ Forced vital capacity ratio

^a^rs9266292 and rs9295950 are in complete LD (r^2^=1) with the top SNP (rs9266290 and rs9295949, respectively)

^b^Multiallelic HLA amino acid polymorphisms

^c^Independent signals identified after the forward-type conditional regression analysis

^d^The frequencies of the alternative alleles in cases and controls (binary traits), and in the overall cohort (quantitative traits) are shown here

^e^The effect size shown here are OR(95% CI) for binary traits and beta value(standard deviation) for quantitative traits

^f^Omnibus test results are shown for the multiallelic HLA amino acid polymorphism, and the test results are shown for the additional independent signals identified after the forward-type conditional regression analysis

^g^References of the previously reported associations are shown in Supplementary table 5

To understand the pattern of associations between HLA variants and phenotypes, we further summarized HLA variants of the GWS association signals by their variation types, genomic locations, and gene classes (Supplementary figure 2). As shown in Supplementary figure 2A, the majority of the GWS signals were biallelic SNVs for most phenotypes. Notably, allelic and amino acid variations were dominant HLA variants in arthritis, T-CHO, and HDL. Furthermore, most of the GWS biallelic SNVs and indels on the HLA region identified in our PheWAS were located on intronic or intergenic regions (Supplementary figure 2B). Finally, these HLA variant-associated phenotypes were categorized into distinct patterns according to the HLA class of their corresponding variants: (1) only class I HLA variations (arthritis, AS, T-CHO, HB, HCT and the VC/HT ratio); (2) only class II HLA variations (drug allergy, asthma, and HDL); (3) both class I and class II HLA variations (TG, creatinine, albumin, SGOT, AFP, RBC, WBC, and platelet) (Supplementary figure 2C).

### Heritability estimates and genetic correlation of HLA variations

**Fig. 5:**
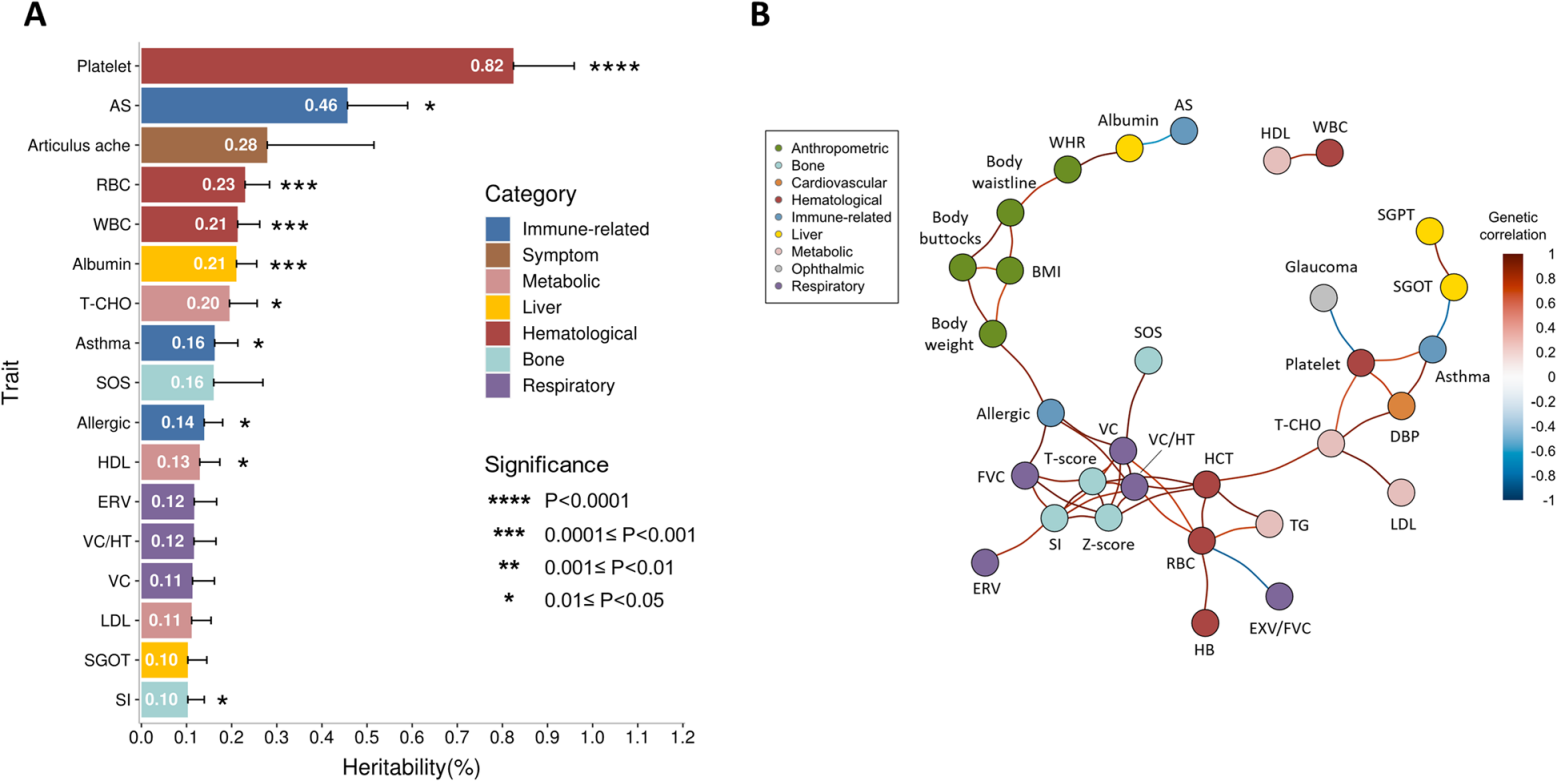
Heritability estimates (**A**) and genetic correlation network (**B**) among 109 phenotypes of HLA polymorphisms. In panel A, only the traits with heritability ≥ 0.10% were shown; in panel B, the traits with *p*-value < 0.05 after FDR correction in the pairwise genetic correlation tests were demonstrated; AS, Ankylosing Spondylitis; RBC, Red blood cell count; WBC, White blood cell count; T-CHO, Total cholesterol; SOS, Speed of sound; HDL, High density lipoprotein cholesterol; ERV, Expiratory reserve volume; VC/HT, Vital capacity/Height ratio; VC, Vital capacity; LDL, Low density lipoprotein cholesterol; SGOT, Serum glutamic oxaloacetic transaminase; SI, Stiffness index; WHR, Waist–hip ratio; BMI, Body mass index; FVC, Forced vital capacity; HB, Hemoglobin; HCT, Hematocrit; EXV/FVC, Extrapolated Volume/ Forced vital capacity ratio; TG, Triglyceride; DBP, Diastolic pressure; SGPT, Serum glutamic pyruvic transaminase

To further understand the genetic effect of the HLA region on each phenotype, we evaluated the phenotypic variances explained by the HLA variations through HLA region-wide heritability estimation. Furthermore, we constructed the HLA region-wide genetic correlation network of phenotypes to discover common HLA genetic features amongst the phenotypes. As shown in Fig. 5A, hematological traits, which exhibited the greatest number of independent signals, had a large proportion of heritability explained by HLA variants. These hematological traits comprised platelet (0.82%), RBC count (0.23%), and WBC count (0.21%). Furthermore, the immune-related traits, including AS (0.46%), asthma (0.16%), and drug allergy (0.14%), also showed high HLA region-wide heritability. Interestingly, several traits without any GWS signals also displayed a large proportion of their variances explained by HLA variants. For example, articulus ache, SOS, and SI showed the HLA region-wide heritability of 0.28%,0.16%, and 0.10%, respectively. In the HLA region-wide genetic correlation network (Fig. 5B), phenotypes were linked together by their shared features in the associations with HLA variants. Generally, phenotypes within the same category were clustered together. For example, WHR, body waistline, body buttocks, body weight, and BMI were all in the cluster of anthropometric phenotypes with strong positive correlations between each other. Furthermore, a cluster of bone-related traits (SI, T-score, and Z-score) and a cluster of liver traits (SGOT and SGPT) were observed. However, there were also some cross-category correlations. For instance, positive correlations between bone density indices and respiratory examinations were detected. Moreover, we found negative correlations between AS and albumin (r_g_= -0.64) and between glaucoma and platelet (r_g_= -0.82).

### Pharmacogenomics of HLA variations

To understand the burden of HLA-related adverse drug reactions (ADR) in the Taiwanese population, we investigated the proportion of carriers of selected HLA alleles with known relationship to ADRs in our HLA- imputed Taiwanese cohort. As shown in Table 2, 30.86% of the imputed cohort were predicted to be HLA-C*01:02 carriers. This allele is known to be associated with higher risk of methazolamide-induced Stevens-Johnson syndrome/toxic epidermal necrolysis (SJS/TEN). Furthermore, approximately 20% of the Taiwanese populations were HLA-B*58:01 carriers, which is associated with various drug-induced severe cutaneous adverse reactions (SCARs).

**Table 2:** Carriers and Frequencies of ADR-related HLA alleles in TWB (*n*=59,448)

Alleles	Carriers (%)	Allele frequencies	Previous Reports of HLA-related ADRs			
			Related-drugs	Reaction	Population	Ref
HLA-C*01:02	21317 (35.86)	0.1991	Methazolamide	SJS/TEN	Korean, Japanese	[36]
HLA-C*03:02	12469 (20.97)	0.1114	Allopurinol	DRESS, SJS/TEN	Korean, Vietnamese	[37, 38]
HLA-B*58:01	12427 (20.9)	0.1110	Allopurinol	SCAR, DRESS, SJS/TEN	Asian, European	[38, 39]
			Carbamazepine	DRESS, SJS/TEN	Asian	[40, 41]
			Lamotrigine	DRESS, SJS/TEN	Multiple ethnicities	[42]
HLA-B*13:01	7133 (12.00)	0.0618	Dapsone	HSS, DRESS, SJS/TEN	Asian	[43–45]
			Phenytoin	SJS/TEN	Asian	[46]
HLA-DPB1*03:01	5502 (9.26)	0.0474	Aspirin	Asthma	Korean	[47]
HLA-B*15:02	4984 (8.38)	0.0428	Carbamazepine	SJS/TEN	Asian	[40, 48, 49]
			Phenytoin	SJS/TEN	Asian	[46, 50–52]
			Lamotrigine	SJS/TEN	Multiple ethnicities	[50, 53, 54]
HLA-B*35:01	3305 (5.56)	0.0282	Nevirapine	HSS, DRESS	Multiple ethnicities	[55, 56]
HLA-A*31:01	2449 (4.12)	0.0208	Carbamazepine	SCAR, DRESS, SJS/TEN	Multiple ethnicities	[49, 57–59]
			Lamotrigine	DRESS, SJS/TEN	Korean	[60]
HLA-B*15:11	944 (1.59)	0.0080	Carbamazepine	DRESS, SJS/TEN	Asian	[41, 61, 62]
HLA-DRB1*01:01	615 (1.03)	0.0052	Nevirapine	DRESS, HSS	Asian, Australian	[55, 63]
HLA-B*57:01	203 (0.34)	0.0017	Abacavir	DRESS, HSS	Multiple ethnicities	[64]
			Flucloxacillin	DILI	European	[65]
HLA-B*59:01	0 (0%)	0.0000	Methazolamide	SJS/TEN	Asian	[66, 67]
			Carbamazepine	SJS/TEN	Japanese	[68]

The carrier numbers and allele frequencies were calculated by the imputed HLA types, *SJS* Steven-Johnson Syndrome, *TEN* Toxic Epidermal Necrolysis, *DRESS* Drug Reaction with Eosinophilia and Systemic Symptoms, *SCAR* Severe cutaneous adverse reaction, *HSS* Hypersensitivity Syndrome, *DILI* Drug-induced liver injury

## Discussion

Here, we constructed an accurate HLA imputation reference panel of eight HLA genes and imputed HLA alleles within the Taiwan Han Chinese population. The HLA allele frequencies are consistent with the previous reports from HLA direct-typing in other Taiwanese cohorts [25–27]. In addition, the haplotype distributions are similar to that in the southern Han Chinese populations [28]. The results indicate the robustness of this reference panel. Furthermore, within a diverse range of phenotypes, we discovered GWS associations with HLA variants, revealing numerous novel findings. Also, we observed both within and across categories correlations amongst the phenotypes accounting for the HLA genetic variations. Finally, the carriers of pharmacogenomic related HLA alleles, including HLA-C*01:02, HLA-B*58:01, and HLA-B*15:02, were characterized in the Taiwanese general population.

Huang et al. nicely constructed a Taiwanese-specific HLA imputation reference panel in 2020 [20]. The imputed HLA allele frequencies in the current study are similar to the findings by Huang et al. However, the imputation accuracies here are slightly better than the previous study (96.8% - 100% compared to 95.9% - 99.8%). Huang et al. utilized the TWBv1 genotype data for reference panel construction, while the reference panel in this study was constructed based on the TWBv2 array genotype. TWBv2 is the latest genome-wide SNP array implemented in the Taiwan biobank project. TWBv2 arrays include more variants across the HLA region [12, 21], which may be attributable to the higher accuracies of the imputation models. In addition, the haplotype frequencies in the TWB HLA imputed cohort are closer to the southern Han Chinese comparing to the other Han Chinese origins [28]. The observations were supported by previous reports that nearly 80% of the TWB subjects are clustered into the southern Han Chinese ancestry. This is also aligned with the immigrant history of Taiwan. The Taiwanese Minnan and Hakka ancestors moved from southeastern coastline of China in the past few centuries [17]. These results indicated the robustness and the reliability of the HLA imputation reference panel constructed in this study.

We explored the phenotypic implications of the HLA genes using the imputed HLA variations, 26 out of 109 examined traits were highlighted as significantly HLA-associated. In addition, a total of 31 independent signals and two variants in complete LD with the top signal were identified. Importantly, the HLA association patterns exhibited a high degree of consistency with previous reports. For example, the significant associations with a multitude of hematological traits, including the RBC, WBC, HB, and HCT, are enriched within the class I HLA genes, especially the *HLA-B* and *HLA-C* genes. Furthermore, the associations with allergic diseases and asthma are concentrated within class I HLA genes. These findings align with the observations in the Japanese and Korean populations [15, 16]. Conversely, we reported novel associations between HLA variations with several phenotypes. For example, in addition to the well-known association between HLA-B*27 and AS [69], our study reveals novel findings of AS association with *HLA-B* pos 138 and *HLA-B*-H138. This indicates a risk effect of histidine at the *HLA-B* position 138 in AS. The other novel associations include the rs9266290 and rs9266292 with drug allergy, and a variety of SNVs with respiratory quantitative traits. This variance could potentially be due to the inconsistency in phenotype collection or the natural differences of HLA genetic architectures amongst distinct ethnicities.

Within the study, we estimated the HLA region-wide heritability for each phenotype. Interestingly, we found that HLA variations only explained less than 1% of the phenotypic variations. This implies the multifactorial nature of phenotypes. The phenotypic variations are attributed to a broader spectrum of genomic variations and the environmental factors. Surprisingly, articulus ache, SOS, and SI were found to have high HLA region-wide heritability. Thus, future study should focus on the associations between HLA variations and these phenotypes in a larger sample size.

In the HLA region-wide genetic correlation analysis, not only within-category correlations but also cross-category correlations were observed. For example, bone density indices are positively correlated with respiratory examinations, which is with the previous findings [70, 71]. Moreover, a negative correlation between AS and albumin was detected. The result was supported by a prior study indicating fibrinogen to albumin ratio as a potential biomarker for AS [72]. Finally, we identified a negative correlation between glaucoma and platelet. The lower serum platelet level and alteration of platelet-related parameters in glaucoma patients imply the platelet activation in the disease progression of glaucoma [73, 74]. Collectively, these findings further demonstrate the pleiotropic feature of the HLA genetic region. Further studies are required to dissect the role of HLA molecules in these cross-category traits.

Given the significant clinical implications of HLA variations in pharmacogenomics, we comprehensively evaluated the burden of ADR-related HLA alleles in the Taiwanese population. In this study, more than twenty percent of the subjects are predicted to carry the HLA-B*58:01 allele, a variant associated with allopurinol-induced SCARs in a variety of Asian and European populations [37–39, 75–79]. Furthermore, approximately eight percent of individuals were identified as HLA-B*15:02 carriers in the Taiwanese population. HLA-B*15:02 allele is associated with a higher risk of carbamazepine-induced SJS/TEN. Importantly, this association has been confirmed in the Asian populations [40, 48–50, 52, 75, 80–84]. Notably, National Health Insurance (NHI) in Taiwan covers the HLA-B*15:02 and HLA-B*58:01 genetic tests before using carbamazepine and allopurinol, respectively [75]. Another finding is that 35.86% of the Taiwanese subjects are predicted to carry HLA-C*01:02, a variant with increased susceptibility to methazolamide-induced SJS/TEN [85, 86]. Both HLA-C*01:02 and HLA-B*59:01 were reported to be associated with methazolamide-induced SJS/TEN. According to previous study, effect size of HLA-C*01:02 on methazolamide-induced SJS/TEN is not as profound as HLA-B*59:01 [67]. However, none of the subjects in the training set was identified to carry HLA-B*59:01. Future studies with rare HLA alleles in the reference panel may improve the imputation accuracy and call rate to identify this allele. Furthermore, previous study indicated multiple factors contributing to pharmacogenomic implications [8]. It will be helpful to investigate the interaction between the cocontributing factors and HLA variants in methazolamide-induced SJS/TEN.

Despite the robust construction of a Taiwanese-specific HLA imputation reference panel and the comprehensively phenotypic exploration of the HLA region, there are still some limitations here. First, the sequencing-based HLA typing data utilized for training the reference panel only contains genotypes of eight classical HLA genes. Therefore, non-classical HLA genes and non-HLA genes in the HLA region cannot be imputed. Second, although the high imputation accuracies were established in our reference panel, the imputed HLA genotypes were not perfect match to the sequencing-based HLA typing. Thus, HLA imputation strategy is useful for the large cohort investigation especially for the phenotype landscape of HLA variations in a population study, while it is not yet an alternative to the clinical sequencing-based HLA typing. On the other hand, the application of this reference panel relies on the existence of alleles in the training set, limiting the imputation for rare HLA alleles. Future studies with an expansion of the sequencing-based HLA typing data will improve the accuracy and call rate of the reference panel. Third, the binary phenotypes analyzed here were collected with self-reported questionnaires, which may lead to a recall bias and data missing . Moreover, the quantitative phenotypes were collected cross-sectionally from the urine and blood tests at subjects' entry to the TWB. Without longitudinal follow up, the biochemical testing may not be able to reflect the long-term health conditions. Finally, ADR information is not available in the TWB, hence, our assessment of ADR risk allele burden is limited to the estimated prevalence of carriers based on the imputation alleles. Combination of genomic data in the TWB and electronic health records of the subjects will provide more information of the phenotypic landscape for HLA variations.

## Conclusion

We successfully developed a Taiwanese-specific HLA imputation reference panel of eight classical HLA genes. This panel is useful to gain insight into the genetic and phenotypic landscape of the HLA variations within the Taiwan Han Chinese population. Importantly, we identified the estimated burden of ADR-related HLA alleles in the Taiwanese population that will be helpful in pharmacogenomic implementation.

### Supplementary Information

**Additional file 1:** **Supplementary figure 1.** Frequencies of the two-, four-, and six-digit HLA alleles before and after HLA imputation. **Supplementary figure 2.** Distribution of variant types, variant annotation types of biallelic SNVs and indels, and HLA classes of the HLA polymorphisms with significant association with phenotypes. **Supplementary table 1.** Classification of the 55 binary traits included in the PheWAS. **Supplementary table 2.** Classification of the 54 quantitative traits included in the PheWAS. **Supplementary table 3.** Overview of common haplotype frequencies in Taiwanese. **Supplementary table 4.** Comparison of haplotype frequencies between Taiwanese and Han Chinese. **Supplementary table 5.** Novelty of the identified independent association signals with genome-wide significance in the entire HLA region.

**Additional file 2:** **Supplementary dataset 1.** HLA allele frequencies in different Taiwanese cohorts.

**Additional file 3.**

## Data Availability

No datasets were generated or analysed during the current study.

## Abbreviations

PheWAS: Phenome-wide association study
ADR: Adverse drug reactions
HLA: Human leukocyte antigen
MHC: Major histocompatibility complex
RA: Rheumatoid arthritis
LD: Linkage disequilibrium
SBT: Sequencing-based typing
TWB: Taiwan biobank
NGS: Next generation sequencing
MAF: Minor allele frequency
HWE: Hardy-Weinberg equilibrium
IBD: Identity by descent
SNVs: Single nucleotide variations
AFND: Allele frequency net database
PCR-SSO: PCR sequence specific oligonucleotide probe
PCR-SSP: PCR sequence specific primer
PCs: Principal components
HE: Haseman-Elston
SJS/TEN: Stevens-Johnson syndrome/toxic epidermal necrolysis
SCARs: Severe cutaneous adverse drug reactions
NHI: National health insurance
AS: Ankylosing spondylitis
QTL: Quantitative trait loci
BMI: Body mass index
WHR: Waist–hip ratio
T-CHO: Total cholesterol
TG: Triglyceride
HDL: High density lipoprotein cholesterol
LDL: Low density lipoprotein cholesterol
SGOT: Serum glutamic oxaloacetic transaminase
SGPT: Serum glutamic pyruvic transaminase
AFP: Alpha-fetoprotein
RBC: Red blood cell count
WBC: White blood cell count
HB: Hemoglobin
HCT: Hematocrit
SI: Stiffness index
SOS: Speed of sound
VC: Vital capacity
IRV: Inspiratory reserve volume
IC: Inspiratory capacity
VC/HT: Vital capacity/height ratio
FVC: Forced vital capacity
FEF75: 75% of Forced expiratory flow
FEF75/HT: 75% of Forced expiratory flow/height ratio
EXV/FVC: Extrapolated volume/forced vital capacity ratio
DRESS: Drug reaction with eosinophilia and systemic symptoms
HSS: Hypersensitivity syndrome
