# Supplementary material for: Phenomic landscape and pharmacogenomic implications for HLA region in a Taiwan Han Chinese population
Source: Biomark Res. 2024 May 3;12:46. doi: 10.1186/s40364-024-00591-z (PMC11067262; doi:10.1186/s40364-024-00591-z)
Supplement: Supplementary file 1 — Additional file 1: Supplementary figure 1. Frequencies of the two-, four-, and six-digit HLA alleles before and after HLA imputation. Supplementary figure 2. Distribution of variant types, variant annotation types of biallelic SNVs and indels, and HLA classes of the HLA polymorphisms with significant association with phenotypes. Supplementary table 1. Classification of the 55 binary traits included in the PheWAS. Supplementary table 2. Classification of the 54 quantitative traits included in the PheWAS. Supplementary table 3. Overview of common haplotype frequencies in Taiwanese. Supplementary table 4. Comparison of haplotype frequencies between Taiwanese and Han Chinese. Supplementary table 5. Novelty of the identified independent association signals with genome-wide significance in the entire HLA region. [file 40364_2024_591_MOESM1_ESM.docx]

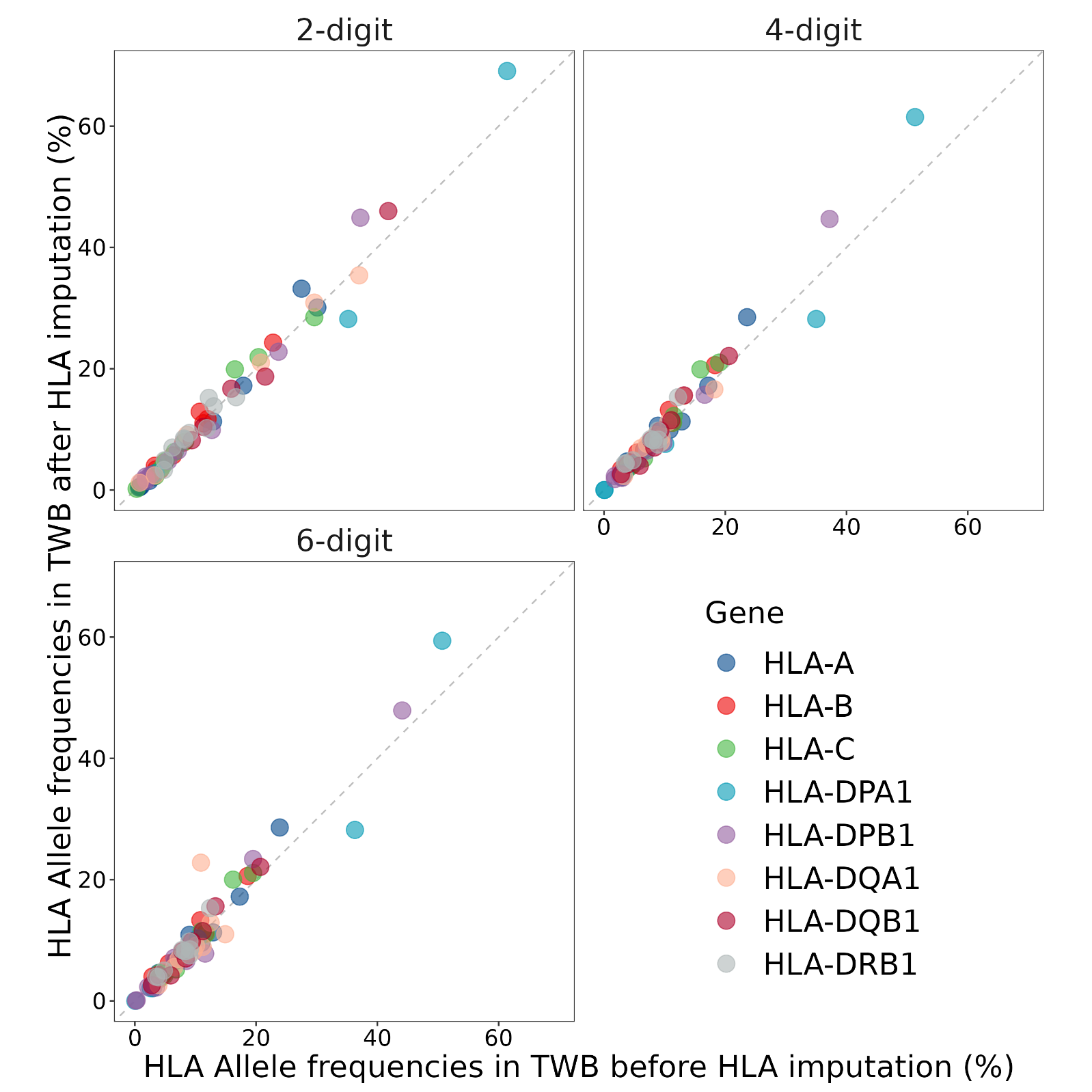


**Supplementary figure 1. Frequencies of the two-, four-, and six-digit HLA alleles before and after HLA imputation.**


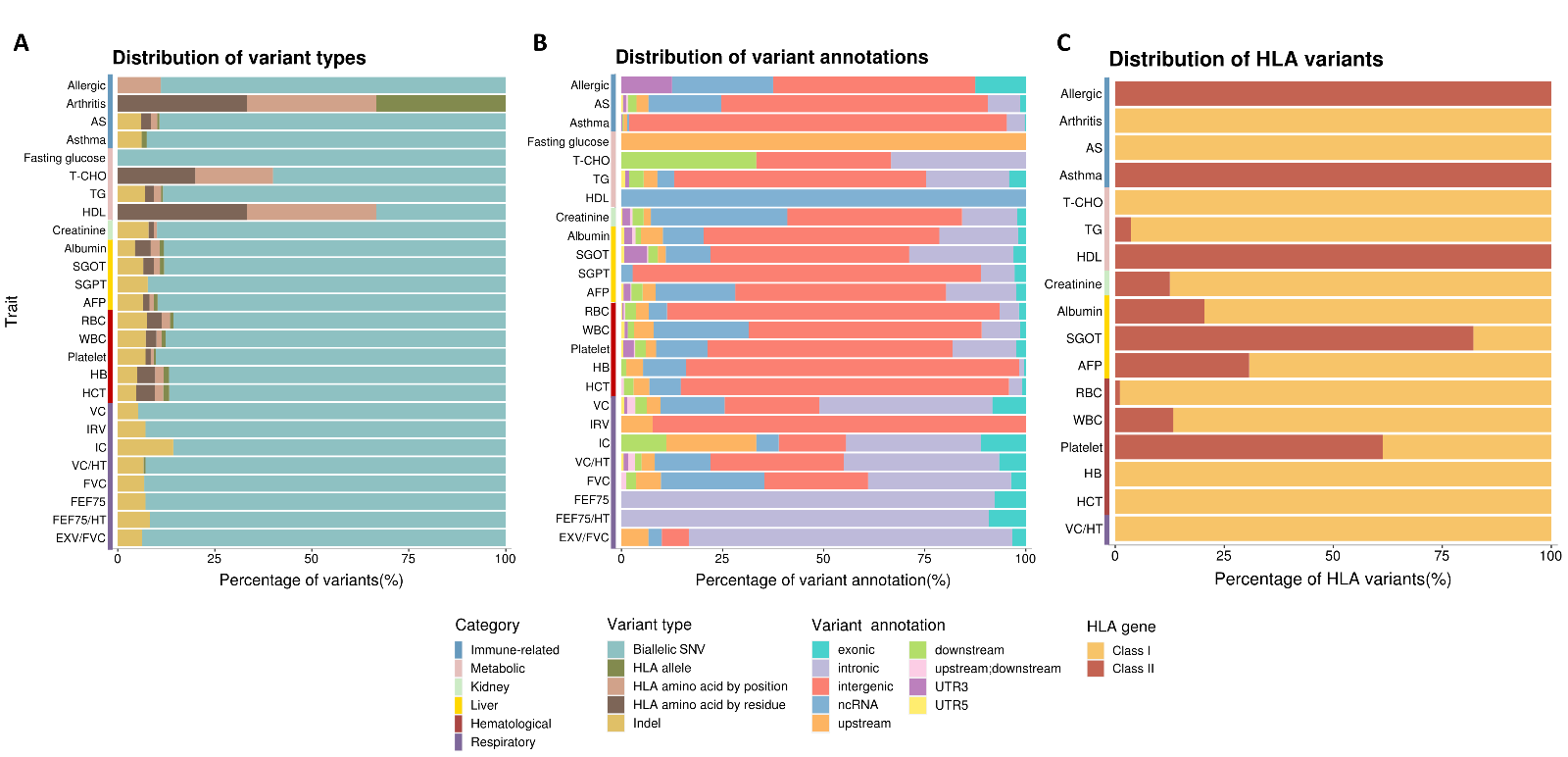


**Supplementary figure 2. Distribution of variant types (A), variant annotation types of biallelic SNVs and indels (B), and HLA classes (C) of the HLA polymorphisms with significant association with phenotypes.** The phenotypes with at least one GWS signal were shown in the plots.

**Supplementary table 1. Classification of the 55 binary traits included in the PheWAS.**

| **Category** | **Trait** | **Cases** | **Controls** | **Total** | **Number of significant variants** |
| --- | --- | --- | --- | --- | --- |
| **Immune-related disease** | Drug allergic (Allergic) | 5739 | 53447 | 59186 | 9 |
|  | Arthritis | 3096 | 56008 | 59104 | 9 |
|  | Rheumatoid arthritis (RA) | 456 | 58648 | 59104 | 0 |
|  | Ankylosing Spondylitis (AS) | 71 | 59033 | 59104 | 1561 |
|  | Gout | 2078 | 57026 | 59104 | 0 |
|  | Asthma | 2100 | 57130 | 59230 | 390 |
| **Metabolic disease** | Valve heart disease | 2498 | 56499 | 58997 | 0 |
|  | Coronary artery disease | 719 | 58278 | 58997 | 0 |
|  | Arrhythmia | 2806 | 56191 | 58997 | 0 |
|  | Cardiomyopathy | 498 | 58499 | 58997 | 0 |
|  | Congenital heart disease | 113 | 58884 | 58997 | 0 |
|  | Hypertension | 7426 | 51571 | 58997 | 0 |
|  | Apoplexia | 374 | 58623 | 58997 | 0 |
| **Cardiovascular disease** | Hyperlipidemia | 4467 | 54530 | 58997 | 0 |
|  | Diabetes | 3092 | 56168 | 59260 | 0 |
|  | Gestational diabetes mellitus | 105 | 59155 | 59260 | 0 |
|  | Type 1 diabetes mellitus | 20 | 59240 | 59260 | 0 |
|  | Type 2 diabetes mellitus | 2996 | 56264 | 59260 | 0 |
| **Kidney disease** | Kidney stone | 3682 | 55333 | 59015 | 0 |
|  | Renal failure | 68 | 58947 | 59015 | 0 |
| **Digestive disease** | Peptic ulcer | 8841 | 50343 | 59184 | 0 |
|  | Gastroesophageal reflux | 8108 | 51076 | 59184 | 0 |
|  | Irritable bowel syndrome | 1453 | 57731 | 59184 | 0 |
|  | Liver gall stone | 2729 | 56286 | 59015 | 0 |
| **Mental disease** | Depression | 2220 | 56878 | 59098 | 0 |
|  | Manic depression | 393 | 58705 | 59098 | 0 |
|  | Obsessive compulsive disease | 49 | 59049 | 59098 | 0 |
|  | Alcoholism drug abuse | 19 | 59079 | 59098 | 0 |
|  | Schizophrenia | 99 | 58999 | 59098 | 0 |
| **Neurologic disease** | Epilepsy | 189 | 59054 | 59243 | 0 |
|  | Hemicrania | 1721 | 57522 | 59243 | 0 |
|  | Multiple sclerosis | 14 | 59229 | 59243 | 0 |
|  | Parkinson | 73 | 59170 | 59243 | 0 |
|  | Dementia | 21 | 59222 | 59243 | 0 |
| **Ophthalmic disease** | Cataract | 5322 | 54125 | 59447 | 0 |
|  | Glaucoma | 806 | 58641 | 59447 | 0 |
|  | Xerophthalmia | 7029 | 52418 | 59447 | 0 |
|  | Retinal detachment | 818 | 58629 | 59447 | 0 |
|  | Floaters | 7482 | 51965 | 59447 | 0 |
|  | Blind | 106 | 59341 | 59447 | 0 |
|  | Color blind | 246 | 59201 | 59447 | 0 |
| **Gynecological disease^a^** | Postpartum depression | 160 | 40175 | 40335 | 0 |
|  | Myoma | 8655 | 31090 | 39745 | 0 |
|  | Ovarian cyst | 2296 | 37356 | 39652 | 0 |
|  | Endometriosis | 2847 | 36825 | 39672 | 0 |
|  | Cervical polyp | 1215 | 38409 | 39624 | 0 |
|  | Dysmenorrhea | 630 | 6395 | 7025 | 0 |
| **Bone disease** | Osteoporosis | 2565 | 56539 | 59104 | 0 |
|  | Degenerative joint disease (DJD) | 2484 | 56620 | 59104 | 0 |
| **Respiratory disease** | Emphysema bronchitis | 687 | 58543 | 59230 | 0 |
| **Symptom** | Vertigo | 3849 | 55166 | 59015 | 0 |
|  | Articulus ache | 2315 | 7091 | 9406 | 0 |
|  | Neck ache | 3565 | 5840 | 9405 | 0 |
|  | Back and waist ache | 3225 | 6180 | 9405 | 0 |
|  | Sciatica | 772 | 8630 | 9402 | 0 |

^a^ Only female controls were included in the gynecological diseases

**Supplementary table 2. Classification of the 54 quantitative traits included in the PheWAS.**

| **Category** | **Trait** | **Total** | **Number of significant variants** |
| --- | --- | --- | --- |
| **Anthropometric QTL** | Body height | 59447 | 0 |
|  | Body weight | 59447 | 0 |
|  | Body fat rate | 57484 | 0 |
|  | Body waistline | 59447 | 0 |
|  | Body buttocks | 59447 | 0 |
|  | Body mass index (BMI) | 59447 | 0 |
|  | Waist–hip ratio (WHR) | 59447 | 0 |
| **Metabolic QTL** | Hemoglobin A1c (HbA1c) | 59448 | 0 |
|  | Fasting glucose | 59448 | 1 |
|  | Total cholesterol (T-CHO) | 59448 | 5 |
|  | Triglyceride (TG) | 59448 | 600 |
|  | High density lipoprotein cholesterol (HDL) | 59448 | 3 |
|  | Low density lipoprotein cholesterol (LDL) | 59448 | 0 |
| **Cardiovascular QTL** | Systolic pressure (SBP) | 59432 | 0 |
|  | Diastolic pressure (DBP) | 59432 | 0 |
|  | Heartbeat speed | 59433 | 0 |
| **Kidney-related QTL** | Blood Urea Nitrogen (BUN) | 59448 | 0 |
|  | Creatinine | 59447 | 366 |
|  | Uric acid | 59448 | 0 |
|  | Microalbumin | 59330 | 0 |
| **Liver-related QTL** | Total bilirubin (TBil) | 59448 | 0 |
|  | Albumin | 59448 | 520 |
|  | Serum glutamic oxaloacetic transaminase (SGOT) | 59448 | 1573 |
|  | Serum glutamic pyruvic transaminase (SGPT) | 59448 | 39 |
|  | γ-Glutamyl transferase (γ-GT) | 59448 | 0 |
|  | Alpha-Fetoprotein (AFP) | 59446 | 2695 |
| **Hematological QTL** | Red blood cell count (RBC) | 59445 | 1419 |
|  | White blood cell count (WBC) | 59445 | 583 |
|  | Platelet | 59444 | 7168 |
|  | Hemoglobin (HB) | 59445 | 280 |
|  | Hematocrit (HCT) | 59445 | 385 |
| **Bone-related QTL** | Stiffness index (SI) | 58459 | 0 |
|  | T-score | 58459 | 0 |
|  | Z-score | 58459 | 0 |
|  | Speed of sound (SOS) | 14523 | 0 |
|  | Broadband ultrasound attenuation (BUA) | 14523 | 0 |
| **Respiratory QTL** | Vital capacity (VC) | 41477 | 153 |
|  | Tidal volume (TV) | 38081 | 0 |
|  | Expiratory reserve volume (ERV) | 38081 | 0 |
|  | Inspiratory Reserve Volume (IRV) | 38080 | 14 |
|  | Inspiratory capacity (IC) | 38081 | 21 |
|  | Vital capacity/Height ratio (VC/HT) | 41477 | 196 |
|  | Forced vital capacity (FVC) | 41478 | 88 |
|  | Forced expiratory volume in 1 second (FEV1) | 41478 | 0 |
|  | FEV1/FVC ratio (%) (FEV1/FVC) | 41478 | 0 |
|  | FEV1/VC ratio (%) (FEV1/VC) | 41469 | 0 |
|  | Forced mid-expiratory flow (FEF25-75%) | 41478 | 0 |
|  | Peak expiratory flow (PEF) | 41478 | 0 |
|  | 25% of Forced expiratory flow (FEF25) | 41478 | 0 |
|  | 50% of Forced expiratory flow (FEF50) | 41478 | 0 |
|  | 75% of Forced expiratory flow (FEF75) | 41478 | 14 |
|  | 75% of Forced expiratory flow/height ratio (FEF75/HT) | 41478 | 12 |
|  | Extrapolated Volume/Forced vital capacity ratio (%) (EXV/FVC) | 41473 | 32 |
|  | Forced inspiratory volume in 1 second/FVC ratio (FIV1/FVC) | 35702 | 0 |

**Supplementary table 3. Self-reported parent originality from subjects in the reference panel (n=845).**

|  |  | Mother | | |
| --- | --- | --- | --- | --- |
|  |  | China | Hakka | Minnan |
| Father | China | 144 | 0 | 8 |
|  | Hakka | 13 | 53 | 18 |
|  | Minnan | 83 | 11 | 508 |

Seven subjects had missing data of self-reported parent originality.

**Supplementary table 4. Comparison of four-digit HLA allele frequencies in TWB before (n=845) and after (n=59448) imputation.**

| gene | statistic (χ^2^) | p-value |
| --- | --- | --- |
| *HLA-A* | 0.038 | 1 |
| *HLA-B* | 0.024 | 1 |
| *HLA-C* | 0.031 | 1 |
| *HLA-DPA1* | 0.044 | 1 |
| *HLA-DPB1* | 0.037 | 1 |
| *HLA-DQA1* | 0.014 | 1 |
| *HLA-DQB1* | 0.021 | 1 |
| *HLA-DRB1* | 0.024 | 1 |

Chi-square goodness of fit tests with the HLA allele frequencies in TWB before imputation (NGS-based HLA typing) as the expected frequencies are applied to test differences in the allele frequencies for each HLA gene before and after HLA imputation.

**Supplementary table 5. Comparison of four-digit HLA allele frequencies in TWB imputed cohort (n=59448) and other Taiwanese cohorts.**

| comparison | gene | statistic (χ^2^) | p-value |
| --- | --- | --- | --- |
| MJ Lai et al. (n=46682) | *HLA-A* | 0.016 | 1 |
|  | *HLA-B* | 0.008 | 1 |
|  | *HLA-DRB1* | 0.016 | 1 |
| MJ Lai et al. aborigines (n=233) | *HLA-A* | 0.597 | 1 |
|  | *HLA-B* | 0.459 | 1 |
|  | *HLA-DRB1* | 0.238 | 1 |
| SH Wen et al. (n=710) | *HLA-A* | 0.006 | 1 |
|  | *HLA-B* | 0.007 | 1 |
|  | *HLA-DRB1* | 0.049 | 1 |
| PL Chen et al. (n=504) | *HLA-A* | 0.062 | 1 |
|  | *HLA-B* | 0.010 | 1 |
|  | *HLA-C* | 0.006 | 1 |
|  | *HLA-DPB1* | 0.034 | 1 |
|  | *HLA-DQB1* | 0.006 | 1 |
|  | *HLA-DRB1* | 0.068 | 1 |

Chi-square goodness of fit tests using the HLA allele frequencies in TWB after imputation as the expected frequencies are applied to test differences of the allele frequencies for each HLA gene in TWB and other Taiwanese cohorts.

**Supplementary table 6. Overview of common haplotype frequencies in Taiwanese.**

| **Haplotypes** | **Frequencies** |
| --- | --- |
| A*33:03-C*03:02-B*58:01-DRB1*03:01-DQA1*05:01-DQB1*02:01-DPA1*01:03-DPB1*04:01 | 0.03853 |
| A*02:07-C*01:02-B*46:01-DRB1*09:01-DQA1*03:02-DQB1*03:03-DPA1*02:02-DPB1*05:01 | 0.0223 |
| A*33:03-C*03:02-B*58:01-DRB1*03:01-DQA1*05:01-DQB1*02:01-DPA1*02:02-DPB1*05:01 | 0.01156 |
| A*02:03-C*07:02-B*38:02-DRB1*16:02-DQA1*01:02-DQB1*05:02-DPA1*02:02-DPB1*05:01 | 0.00945 |
| A*33:03-C*03:02-B*58:01-DRB1*13:02-DQA1*01:02-DQB1*06:09-DPA1*02:01-DPB1*09:01 | 0.00797 |
| A*02:01-C*15:02-B*40:01-DRB1*11:01-DQA1*05:05-DQB1*03:01-DPA1*02:02-DPB1*05:01 | 0.00758 |
| A*11:01-C*01:02-B*46:01-DRB1*09:01-DQA1*03:02-DQB1*03:03-DPA1*02:02-DPB1*05:01 | 0.00695 |
| A*11:01-C*07:02-B*40:01-DRB1*09:01-DQA1*03:02-DQB1*03:03-DPA1*02:02-DPB1*05:01 | 0.00679 |
| A*11:01-C*08:01-B*15:02-DRB1*12:02-DQA1*06:01-DQB1*03:01-DPA1*01:03-DPB1*21:01 | 0.00671 |
| A*30:01-C*06:02-B*13:02-DRB1*07:01-DQA1*02:01-DQB1*02:02-DPA1*02:01-DPB1*17:01 | 0.00618 |
| A*02:07-C*01:02-B*46:01-DRB1*08:03-DQA1*01:03-DQB1*06:01-DPA1*02:02-DPB1*05:01 | 0.00579 |
| A*11:01-C*07:02-B*40:01-DRB1*08:03-DQA1*01:03-DQB1*06:01-DPA1*02:02-DPB1*05:01 | 0.00535 |
| A*11:01-C*08:01-B*15:02-DRB1*12:02-DQA1*06:01-DQB1*03:01-DPA1*02:02-DPB1*05:01 | 0.00506 |
| A*02:07-C*01:02-B*46:01-DRB1*09:01-DQA1*03:02-DQB1*03:03-DPA1*04:01-DPB1*13:01 | 0.00503 |

Common haplotypes in the Taiwanese population were defined as frequency > 0.005.

**Supplementary table 7. Comparison of haplotype frequencies between Taiwanese and Han Chinese.**

| **Haplotypes** | **Frequencies** | | | | | |
| --- | --- | --- | --- | --- | --- | --- |
|  | **Before HLA Imputation (n=845)** | **After HLA Imputation (n=59448)** | **Overall HAN^a^** | **Northern HAN^a^** | **Central HAN^a^** | **Southern HAN^a^** |
| A*30:01-C*06:02-B*13:02-DRB1*07:01-DQB1*02:02 | 0.0232 | 0.0115 | 0.0389 | 0.045-0.050 | 0.035-0.040 | 0.015-0.020 |
| A*02:07-C*01:02-B*46:01-DRB1*09:01-DQB1*03:03 | 0.0336 | 0.038 | 0.0213 | 0.015-0.020 | 0.025-0.030 | 0.030-0.035 |
| A*33:03-C*03:02-B*58:01-DRB1*03:01-DQB1*02:01 | 0.0713 | 0.0585 | 0.0174 | 0.010-0.015 | 0.015-0.020 | 0.030-0.035 |
| A*33:03-C*03:02-B*58:01-DRB1*13:02-DQB1*06:09 | 0.0257 | 0.017 | 0.0106 | 0.005-0.010 | 0.010-0.015 | 0.005-0.010 |
| A*02:07-C*01:02-B*46:01-DRB1*08:03-DQB1*06:01 | 0.0108 | 0.0108 | 0.0086 | 0.005-0.010 | 0.010-0.015 | 0.005-0.010 |
| A*33:03-C*14:03-B*44:03-DRB1*13:02-DQB1*06:04 | 0.006 | 0.0008 | 0.0085 | 0.005-0.010 | 0.010-0.015 | 0.000-0.005 |
| A*02:01-C*03:04-B*13:01-DRB1*12:02-DQB1*03:01 | 0.006 | 0.004 | 0.0083 | 0.010-0.015 | 0.005-0.010 | 0.005-0.010 |
| A*11:01-C*08:01-B*15:02-DRB1*12:02-DQB1*03:01 | 0.0093 | 0.0149 | 0.0078 | 0.005-0.010 | 0.005-0.010 | 0.015-0.020 |
| A*11:01-C*04:01-B*15:01-DRB1*04:06-DQB1*03:02 | 0.0034 | 0.0062 | 0.0065 | 0.000-0.005 | 0.005-0.010 | 0.005-0.010 |
| A*01:01-C*06:02-B*37:01-DRB1*10:01-DQB1*05:01 | 0 | 0.0019 | 0.0063 | 0.005-0.010 | 0.005-0.010 | 0.005-0.010 |
| A*02:01-C*03:03-B*15:11-DRB1*09:01-DQB1*03:03 | 0.0046 | 0.002 | 0.0056 | 0.005-0.010 | 0.005-0.010 | 0.000-0.005 |
| A*33:03-C*07:06-B*44:03-DRB1*07:01-DQB1*02:02 | 0.0034 | 0.0013 | 0.0055 | 0.005-0.010 | 0.005-0.010 | 0.000-0.005 |

^a^ HAN, Han Chinese

**Supplementary table 8. Novelty of the identified independent association signals with genome-wide significance in the entire HLA region.**

| **Category** | **Traits** | **Variants** | **Position (hg38)** | **Gene** | **Previous reported** | **PubMed ID** |
| --- | --- | --- | --- | --- | --- | --- |
| Immune-related disease | Allergic | rs9266290 | 31361306 | *HLA-B* | No | - |
|  |  | rs9266292^a^ | 31361316 | *HLA-B* | No | - |
|  | Arthritis | *HLA-B**27:04:01 | - | *HLA-B* | No | 24062861 |
|  | Asthma | *HLA-DQA1**06:01 | - | *HLA-DQA1* | Yes | 11802952 |
|  | AS | *HLA-B* pos 138^b^ | - | *HLA-B* | No | - |
| Metabolic QTL | Fasting glucose | rs2074489 | 31272351 | *HLA-C (upstream)* | No | - |
|  | HDL | *HLA-DRB1*-L92 | - | *HLA-DRB1* | No | - |
|  | T-CHO | *HLA-B*-Q94 | - | *HLA-B* | No | - |
|  | TG | rs3873333 | 30928275 | *VARS2 (downstream)* | Yes | 29507422,32203549,32154731,20686565,33339817 |
| Kidney-related QTL | Creatinine | rs2853941 | 31281452 | *RPL3P2* | Yes | 29403010 |
| Liver-related QTL | Albumin | rs6919086 | 31335150 | *-* | Yes | 29403010,34594039 |
|  | SGOT | rs78110044 | 32559333 | *HLA-DRB6* | Yes | 29403010,34594039,33339817,33547301 |
|  | SGPT | rs76089289 | 32997926 | *-* | Yes | 34315874,34594039,33547301 |
|  | AFP | *HLA-C*-Y140 | - | *HLA-C* | No | - |
| Hematological QTL | RBC | rs138428160 | 31267408 | *HLA-C (downstream)* | Yes | 27863252,32888493,32888494 |
|  |  | *HLA-A*-V100^c^ | - | *HLA-A* | No | - |
|  | WBC | *HLA-C* pos 140^c^ | - | *HLA-C* | No | - |
|  |  | rs1056429^c^ | 31354106 | *HLA-B* | Yes | 28158719,27863252,29403010,25096241,20139978,21738480,31217584,34107879,34594039,32888494 |
|  | Platelet | rs4713574 | 32659261 | *HLA-DQB1 (downstream)* | Yes | 22139419,34469753 |
|  |  | rs9394145^c^ | 33432001 | *SYNGAP1* | Yes | 26805783,27863252,28031487,29066854,25705162,23263863,20139978,22423221,22139419,24026423,33545615,34469753,32888493,31217584,34107879,34594039,32888494 |
|  |  | rs3131002^c^ | 31124892 | *PSORS1C1* | Yes | 27863252,29403010,22139419,34469753,32888494 |
|  |  | rs3094575^c^ | 29548025 | *OR2I1P* | Yes | 26805783,27863252,31217584,32888493 |
|  | HB | rs3134768 | 31239067 | *-* | Yes | 27863252,32327693,29403010,32888494,34594039 |
|  | HCT | rs3132521 | 31234903 | *-* | Yes | 32888494,32888493,34594039 |
| Respiratory QTL | VC | rs41268928 | 32179380 | *RNF5* | No | - |
|  | IC | rs41268928 | 32179380 | *RNF5* | No | - |
|  | VC/HT | rs41268928 | 32179380 | *RNF5* | No | - |
|  | FVC | rs41268928 | 32179380 | *RNF5* | Yes | 30804560,33766948 |
|  | IRV | rs9295949 | 31078024 | *RNU6-1133P* | No | - |
|  |  | rs9295950 | 31078025 | *RNU6-1133P* | No | - |
|  | EXV/FVC | rs2070600 | 32183666 | *AGER* | No | - |
|  | FEF75 | rs2022059 | 32188712 | *PBX2* | No | - |
|  | FEF75/HT | rs2022059 | 32188712 | *PBX2* | No | - |

^a^rs9266292 and rs9295950 are in complete LD (r^2^=1) with the top SNP (rs9266290 and rs9295949, correspondingly); ^b^Multiallelic HLA amino acid polymorphisms; ^c^Independent signals identified after the forward-type conditional regression analysis; AS, Ankylosing Spondylitis; T-CHO, Total cholesterol; TG, Triglyceride; HDL, High density lipoprotein cholesterol; SGOT, Serum glutamic oxaloacetic transaminase; SGPT, Serum glutamic pyruvic transaminase; AFP, Alpha-Fetoprotein; RBC, Red blood cell count; WBC, White blood cell count; HB, Hemoglobin; HCT, Hematocrit; VC, Vital capacity; IRV, Inspiratory Reserve Volume; IC, Inspiratory capacity; VC/HT, Vital capacity/Height ratio; FVC, Forced vital capacity; FEF75, 75% of Forced expiratory flow; FEF75/HT, 75% of Forced expiratory flow/height ratio; EXV/FVC, Extrapolated Volume/ Forced vital capacity ratio.
